# Semi-automatic extraction of liana stems from terrestrial LiDAR point clouds of tropical rainforests

**DOI:** 10.1016/j.isprsjprs.2019.05.011

**Published:** 2019-08

**Authors:** Sruthi M. Krishna Moorthy, Yunfei Bao, Kim Calders, Stefan A. Schnitzer, Hans Verbeeck

**Affiliations:** a CAVElab – Computational and Applied Vegetation Ecology, Ghent University, 9000 Ghent, Belgium; b Beijing Institute of Space Mechanics and Electricity, No. 104, Road Youyi, Beijing 100094, China; c Department of Biological Sciences, Marquette University, Milwaukee, WI 53201-1881, USA; d Smithsonian Tropical Research Institute, Apartado 0843-03092, Balboa, Ancon, Panama

**Keywords:** Automated liana extraction, Lianas, Terrestial LiDAR, Machine learning, Python package, Tropical forests

## Abstract

Lianas are key structural elements of tropical forests having a large impact on the global carbon cycle by reducing tree growth and increasing tree mortality. Despite the reported increasing abundance of lianas across neotropics, very few studies have attempted to quantify the impact of lianas on tree and forest structure. Recent advances in high resolution terrestrial laser scanning (TLS) systems have enabled us to quantify the forest structure, in an unprecedented detail. However, the uptake of TLS technology to study lianas has not kept up with the same pace as it has for trees. The slower technological adoption of TLS to study lianas is due to the lack of methods to study these complex growth forms. In this study, we present a semi-automatic method to extract liana woody components from plot-level TLS data of a tropical rainforest. We tested the method in eight plots from two different tropical rainforest sites (two in Gigante Peninsula, Panama and six in Nouragues, French Guiana) along an increasing gradient of liana infestation (from plots with low liana density to plots with very high liana density). Our method uses a machine learning model based on the Random Forest (RF) algorithm. The RF algorithm is trained on the eigen features extracted from the points in 3D at multiple spatial scales. The RF based liana stem extraction method successfully extracts on average 58% of liana woody points in our dataset with a high precision of 88%. We also present simple post-processing steps that increase the percentage of extracted liana stems from 54% to 90% in Nouragues and 65% to 70% in Gigante Peninsula without compromising on the precision. We provide the entire processing pipeline as an open source python package. Our method will facilitate new research to study lianas as it enables the monitoring of liana abundance, growth and biomass in forest plots. In addition, the method facilitates the easier processing of 3D data to study tree structure from a liana-infested forest.

## Highlights

• Semi-automated method to extract liana woody points from terrestrial LiDAR data.
• Lianas woody points are classified by a machine learning model.
• Post-processing steps improve the classification performance from 60% to 80%.
• Validation on two tropical forest sites indicates successful liana extraction.

## Graphical abstract

Illustrating the different spatial distribution of points in local spatial scale for (a) liana, (b) tree and (c) leaf points. The local geometry is calculated by the Eigen vectors for the set of points indicated by black squares from a tropical forest plot in Nouragues, French Guiana. $\lambda_{1}$, $\lambda_{2}$ and $\lambda_{3}$ indicate the corresponding normalized eigen values.

**Figure:**
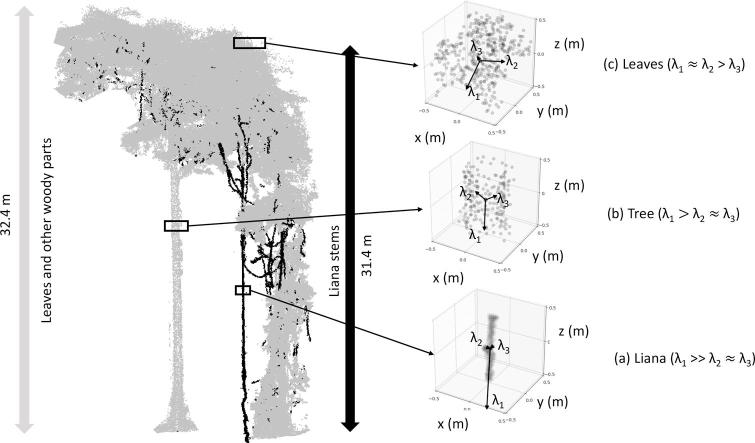

## 1 Introduction

Tropical forests are undergoing large-scale structural changes including an increase in liana abundance and biomass (Schnitzer and Bongers, 2011). Lianas are woody climbing plants that use trees as structural support for ascending to the canopy (Schnitzer and Bongers, 2002) and compete with trees for both aboveground and belowground resources (Toledo-Aceves, 2015, Rodríguez-Ronderos et al., 2016). Lianas have shown to reduce the net above-carbon uptake of the forest by ≈ 76% in three years mostly by reducing tree growth (van der Heijden et al., 2015). As a result, increase in liana abundance results in reduced tree growth, survival, reproduction, biomass and increased tree mortality thereby playing an important role in the global carbon cycle (Schnitzer, 2018, García et al., 2018, van der Heijden et al., 2015).

Attempts to disentangle aboveground and belowground competition between lianas and trees have shown that aboveground competition affected the allocation of biomass in trees and resulted in poorly developed crowns (Schnitzer, 2005). A recent study has shown that liana load on trees can alter the allometry of trees by decreasing tree slenderness resulting in shorter and thicker stems (Dias et al., 2017).

Recent advances in remote sensing technologies have enabled us to view the forest structural complexity in new and unprecedented ways. Terrestrial laser scanning (TLS) is an active remote sensing technique and can measure various forest structural parameters non-destructively with high spatial accuracy (Danson et al., 2007, Jupp et al., 2009, Calders et al., 2014, Calders et al., 2015). Because of the millimeter-level spatial resolution, TLS is potentially useful to study the plot-level impact of lianas on forest structure. In the past decade, there have been significant advancements in the methods developed for 3D point cloud classification using machine learning (Brodu and Lague, 2012). In particular, progress has been made to separate leaf and wood points from 3D data of forests using supervised and/or unsupervised machine learning algorithms. The methods developed were either based on the radiometric features Béland et al. (2014) or geometric features Ma et al., 2016, Wang et al., 2017, Yun et al., 2016, Belton et al., 2013, Wang et al., 2018, Vicari et al., 2019 or a combination of both Zhu et al. (2018).

Despite the increasing importance of lianas, very few studies have attempted to quantitatively study the liana structure and the impact of lianas on individual tree and forest structure using TLS or other such remote sensing technologies (Rodríguez-Ronderos et al., 2016, Sánchez-Azofeifa et al., 2015, Sánchez-Azofeifa et al., 2017, Krishna Moorthy et al., 2018). A recent study has attempted to classify liana stems using a machine learning algorithm in a point cloud using intensity values from the sensor and geometric features, which makes the proposed method sensor-specific (Bao et al., 2018). One of the main reasons for the slower uptake of TLS for studying lianas is that, unlike trees, lianas have irregular growth forms making them harder to study (Bongers et al., 2005). There are multiple reasons why the existing tree segmentation algorithms (Burt et al., 2018, Hackenberg et al., 2015) or machine learning-based leaf vs wood segmentation algorithms (Ma et al., 2016, Wang et al., 2017) do not work for segmenting or identifying lianas from a plot. The main reasons are listed as follows:

• lianas are in general very small with almost all of the stems falling within 10 cm diameter range. (Dewalt et al., 2000, Phillips et al., 2005)
• unlike trees, lianas are generally not self-supporting and hence are generally thinner, longer and have irregular growth patterns. Our visual observations in the field show that they can extend in almost all the directions and tend to have branches that loop to the ground multiple times before shooting up to the canopy.
• unlike trees, lianas deploy their leaves in the mid- and upper-crown region of their host trees thus avoiding the need for a tree crown-like structure (Rodríguez-Ronderos et al., 2016, Krishna Moorthy et al., 2018)

The main objective of the study is to build a novel machine-learning based method for extracting liana woody points from 3D point clouds of tropical rainforests, based only on the geometrical features extracted from the points. The method builds on and extends the existing approaches for 3D point cloud classification, especially that of (Brodu and Lague, 2012). In this manuscript, we explain in detail the TLS data used for developing and testing the method (Section 2), entire processing pipeline for liana stem point extraction (Section 3), critical evaluation of the proposed approach on field data from two rainforest sites (Section 4). In addition, we provide the entire processing pipeline for liana woody point extraction as an open source python package.

With increasing liana abundance across pan-tropical regions, the presented method will accelerate the research to study lianas with data from remote sensing platforms like TLS (Phillips et al., 2002, Campbell et al., 2018). By extracting liana stems from 3D point clouds, the method provides an easier and consistent way to monitor liana stem abundance and potentially stem biomass over time in forest plots. In addition, the method would enable the easy processing of 3D data to study trees from liana-rich sites.

## 2 Study area and LiDAR data collection

We collected TLS data from multiple plots of two different tropical forest sites: Gigante Peninsula, Panama (Feb - Apr, 2016) and Nouragues, French Guiana (August - October, 2017). Gigante Peninsula is a ≈ 60-year old secondary seasonal tropical forest with high liana density (2544 lianas ⩾1cm per ha) (van der Heijden et al., 2015). Nouragues is an undisturbed old-growth lowland moist tropical forest with medium liana density (1200 lianas ⩾1cm per ha) (Schnitzer and DeWalt, 2006).

On Gigante Peninsula, we collected data from the centre 15 m × 15 m area of two plots of a long-term liana removal experiment, where in eight out of the sixteen plots lianas were cut since 2011 (van der Heijden et al., 2015). TLS data was collected from four scan locations at the corners of the two 15 m × 15 m plots. In Nouragues, TLS data was collected by following a radial pattern around six trees with the trees of interest at the centre. The location of the scan positions were chosen in such a way as to get better visibility of the central tree and the surrounding lianas. We randomly distributed 20 to 35 retro-reflective targets in the field to co-register the data from all the scan locations to get a single high resolution 3D point cloud with minimal occlusion (Wilkes et al., 2017). We have attached the scanning pattern for each of the six plots in Nouragues in Appendix A.1.

All the data were collected either using RIEGL VZ-400 (Gigante Peninsula) or RIEGL VZ-1000 (Nouragues) scanners (RIEGL Laser Measurement Systems GmbH, Horn, Austria). Both are multiple return time-of-flight based scanners using a narrow infrared laser beam of wavelength 1550 nm and a beam divergence of 0.35 mrad. Based on the manufacturer’s specifications, the data from the two scanners are interoperable (Calders et al., 2017). The registration of data from all scan positions for each plot was done using the RISCAN Pro software (version 2.5.3, RIEGL Laser Measurement Systems GmbH, Horn, Austria) provided by RIEGL. Detailed description of the number of plots per site, plot size, liana and tree density of the plots, proportion of liana points, the TLS scanner and the angular resolution used for the specific plot is given in Table 1.

**Table 1:** Description of the plots where the TLS data were collected.

Site	Plot Name	Plot size	Liana stems	Tree stems	Liana points proportion (%)	Scanner used	No. of scans per plot	Angular resolution used (deg)
Gigante Peninsula, Panama	GIG_1	15 m by 15 m	56	18	8	RIEGL VZ-400	4	0.02
	GIG_2		46	9	6			

Nouragues, French Guiana	NOU_1	10 m (circle)	1	11	3	RIEGL VZ-1000	3	0.04
	NOU_2	10 m (circle)	1	11	4		3	
	NOU_3	15 m (circle)	3	22	1		6	
	NOU_4	15 m (circle)	2	19	6		5	
	NOU_5	15 m (circle)	3	26	3		5	
	NOU_6	10 m (circle)	1	14	2		4	

## 3 Liana stem extraction pipeline

**Fig. 1:**
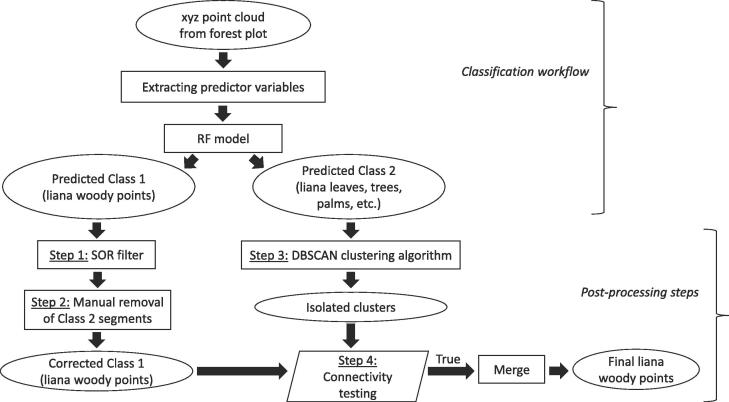
Pipeline for extracting lianas from new forest plots.

In this section, we explain in detail about the RF model built to classify the liana points. We also outline the workflow to use the trained RF model to extract lianas from new previously unseen forest plots as shown in Fig. 1.

### 3.1 Random Forest model development

This section explains in detail all the steps involved in building the RF model for liana woody point classification.

#### 3.1.1 Classification problem

We define the extraction of liana woody points from 3D point clouds of tropical rainforest plots as a binary classification problem. We define the two classes as follows:

• **Class 1** refers to liana woody components in the forest plot
• **Class 2** refers to other vegetation in the forest plots, including but not limited to liana leaves, tree stems and leaves, palms, etc.

#### 3.1.2 Training and validation data preparation

The first step to build an automated liana extraction model using a supervised machine learning classifier is to have labelled training data from which a classifier can learn. The data should be as representative of the real problem as possible to create a robust classifier. The co-registered TLS data from the two plots (plots GIG_1 and GIG_2) of Gigante Peninsula and the six plots from Nouragues were manually labelled into two different classes: 1 being liana woody points and 2 being non-liana points (including liana leaves, trees, etc.).

The manual labelling was the most intensive part of the machine learning pipeline. We labelled the liana woody points in the tree crowns as far as we could visually follow them or up until the liana stems started to branch out to leaves. In dense canopies, it was indeed difficult to follow them all the way up to the top owing to occlusion and the sensor limitation to resolve an object of smaller size at increasing distance from the LiDAR. The manual labelling was done in CloudCompare by an experienced person and took almost 120 man-hours (version 2.8.1, CloudCompare, GPL software) (Girardeau-Montaut, 2011).

After manual labelling, we downsampled the TLS data using a voxel grid filter of size 0.04 m. We chose 0.04 m as the size for voxel grid filter to make a trade-off between retaining the important information in the point cloud and reducing the computational complexity (Burt et al., 2018). Downsampling ensures a uniform distribution of points and aids faster computation time by reducing the number of redundant points, especially the points closer to the scanner. Though it is not absolutely necessary to perform downsampling, it is recommended as it would improve the computational and memory efficiency of the presented algorithm by few-folds. However, the voxel grid size should be chosen based on the problem at hand and the beam divergence of the scanner used.

In liana woody point classification, the classes are imbalanced with liana woody points being the minority class contributing only to an average of ≈ 5% of all the points in a plot. To improve the performance of the machine learning classifier when liana woody points represent < 5% of all points in a plot (eg. NOU_3 in Table 1), we randomly under sampled 10% of the majority class to alter the class distribution (Van Hulse et al., 2007).

**Fig. 2:**
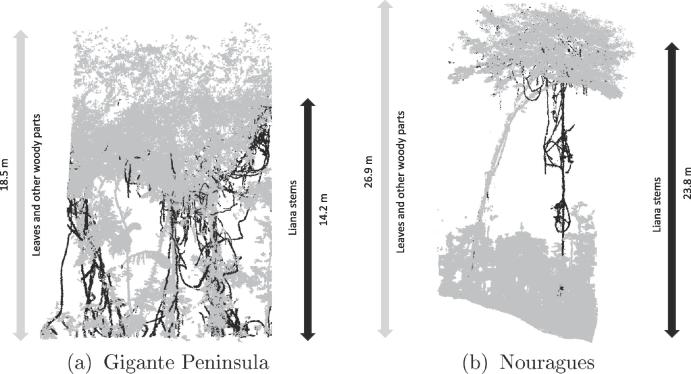
Sample training data from two different sites (a) plot GIG_2 from Gigante Peninsula and (b) plot NOU_4 from Nouragues.

Since our dataset is limited, we used all the points for training and validation of the model. Sample training data from these two different sites are shown in Fig. 2.

#### 3.1.3 Predictor variables

In this study, we use the normalized eigen values of every point in the TLS data from a plot, across multiple spatial scales, as the predictor variables to classify liana woody points. We base our idea on the work of Brodu and Lague (2012), who used eigen values to determine the local dimensionality at multiple scales of a point in 3D space to separate riparian vegetation from ground. Previous studies have used eigen values from TLS data at single spatial scale in forest ecosystems to separate leaf from wood points, where the choice of the right spatial scale was domain-specific (Ma et al., 2016, Wang et al., 2017). Few other studies have proposed to choose the optimal spatial scale from multiple scales for leaf-wood classification (Zhu et al., 2018). Here, we show that the same concept of eigen values, but combined at multiple scales, is effective in highly complex natural scenes like tropical forests to separate liana growth forms from the rest.

The following are the steps involved in the extraction of normalized eigen values from the point cloud:

1. to obtain eigen values at multiple spatial scales, we defined a sphere around each point with that point at the centre with the following radii: 0.1, 0.25, 0.5, 0.75 and 1.0 m. The point at the centre of the sphere is referred to as the target point and all the points within the sphere are considered as the neighbors for the target point. These neighbors at multiple scales define the local dimensionality of the target point at that particular scale. We did not consider spheres with radii below 0.1 m, as our voxel grid based downsampling of size 0.04 m will render most of the points without sufficient neighbors. Also we did not go beyond 1 m for the practical purpose of avoiding high memory requirements.
2. we computed the covariance matrix of the target point along with its neighbors at the above-mentioned spatial scales. As a result, we had five 3 × 3 covariance matrices for each point. The covariance matrix defines the spread and orientation of the points at that scale.
3. we computed the eigen values and vectors of the covariance matrix at each scale for each of the points. This means that we have 3 eigen vectors corresponding to three eigen values per point with the largest eigenvector pointing in the direction of largest variance of the data and the corresponding eigenvalue indicating the magnitude. With three eigen values for each of the five covariance matrices, we had a total of 15 eigen values for each point.
4. to standardize all eigen values into a common range, we normalized the eigen values of each point for each scale between 0 and 1. This was simply done by dividing each eigenvalue by the sum of all three eigen values (denoted as $\lambda_{1},\lambda_{2},\lambda_{3}$, (x m) where $\lambda_{1}$>$\lambda_{2}$>$\lambda_{3}$ and x ∈ {0.1, 0.25, 0.5, 0.75, 1.0}). As a result, we had 15 normalized eigen values for the five chosen scales of interest.

**Fig. 3:**
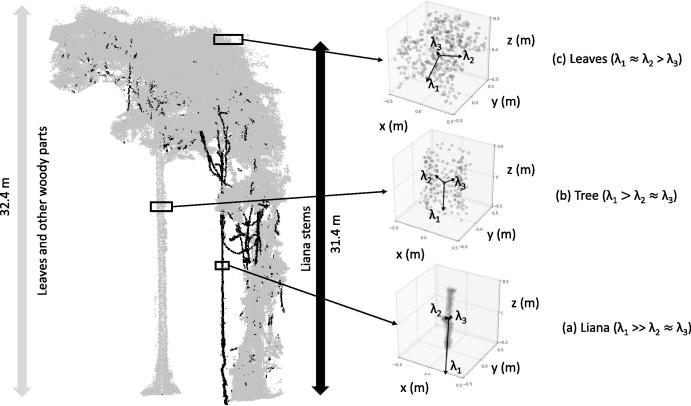
Eigen vectors calculated for a set of (a) liana, (b) tree and (c) leaf points indicated by black squares from plot NOU_5. $\lambda_{1},\lambda_{2}$ and $\lambda_{3}$ indicate the corresponding normalized eigen values.

Fig. 3 illustrates the eigen vectors calculated for a set of tree, liana and leaf points.

**Fig. 4:**
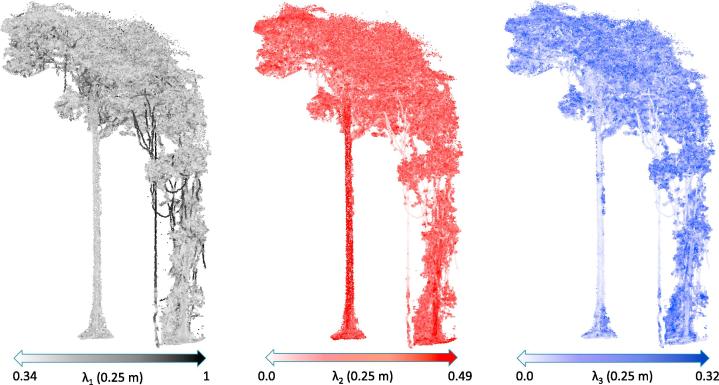
Illustrating the effectiveness of eigen values for classifying lianas in Nouragues (sample from plot NOU_5 in Table 1). The ground truth of the same plot is given in Fig. 3. The color scale at the bottom indicate the range of $\lambda_{1},\lambda_{2}$ and $\lambda_{3}$ at 0.25 m spatial scale from left to rig.ht respectively. (For interpretation of the references to colour in this figure legend, the reader is referred to the web version of this article.)

Since lianas are unique in a way that they are very small compared to their counterparts across all vertical heights and do not usually branch out to leaves unless they are in the mid- or upper-crown region of the host trees, their eigen values represent a dominant variance across one direction at multiple spatial scales (indicated by very high $\lambda_{1}$ at 0.25 m as shown in Fig. 4. The eigen values $\lambda_{1},\lambda_{2}$ and $\lambda_{3}$ at all the spatial scales are shown in the Appendix A.2). Using multiple spatial scales has some clear advantages, as some of the small branches in the understory or canopy might have a dominant variance in one direction at one spatial scale and probably not at the other.

#### 3.1.4 Building the RF model

We selected the Random Forest (RF) machine learning classifier to build the model for the automated liana woody points extraction algorithm. The RF has been widely used in different fields including remote sensing mainly because of its ability to limit overfitting without compromising on the accuracy of the classifier. The algorithm achieves this ability by training random subsets of the data and random subsets of the features for each decision tree. In addition, the RF has been proved when using TLS point clouds as the most robust learner to separate photosynthetic vs non-photosynthetic elements in forest ecosystems (Wang et al., 2017). Since our problem is a binary classification problem (liana stem points vs leaves and other non-liana points) in a similar ecosystem, we chose the RF algorithm to build the classifier. For more details on RF classifier, we refer to the original paper of Breiman (2001).

There are two important hyperparameters to be specified when employing RF for training: number of decision trees to be constructed and number of random features to be chosen while constructing a tree. We performed an exhaustive grid search with the following parameter values for estimating the best combination of these two hyperparameters: Number of decision trees: 10, 20, …100 and Number of random features per decision tree: 2, 3, …8.

The model performance for the hyperparameter optimization was assessed by k-fold cross-validation approach and the model that had the best performance was chosen. The performance metrics used for the assessment are described in the following Section 3.1.5.

Once the hyperparameters are optimized, we built the final model on the entire dataset (all eight plots from two sites indicated in Table 1). However, since our dataset is quite limited, we assessed the performance of the model via k-fold spatial cross-validation, thus eliminating the need for an independent test set.

#### 3.1.5 Assessing the predictive performance

The performance of the liana woody points classification model is assessed by k-fold spatial cross-validation approach (Goetz et al., 2015). This ensures that the model’s fit on the dataset that is independent of the dataset used for training is estimated, while still using all the available information for training. In normal k-fold cross-validation, the data is randomly split into k disjoint sets. Out of the k sets, one set at a time is used for validating the model and the other k-1 sets are used for training. The model performance is then reported as the average performance of the k different model runs. In k-fold spatial cross-validation approach, instead of splitting the data into k random sub-sets, we split the data into k spatially disjoint sub-areas.

We evaluate the model performance in the following three different categories:

1. **self-site spatial cross-validation,** where the models are trained and validated on the same site. For Gigante site, we split the two 15 by 15 m plots into four equal disjoint sub-areas of size 7.5 by 7.5 m each and performed a eightfold spatial cross-validation as shown in Fig. 5. Since the plots in Nouraugues had only one to three liana individuals in a plot, we simply performed a sixfold cross-validation with one plot retained for testing and the rest of the five plots used for training.

**Fig. 5:**
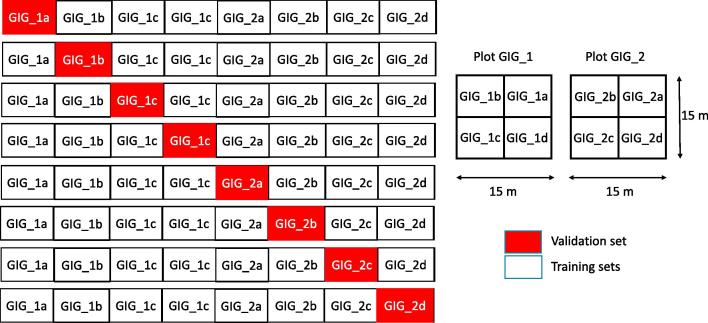
Eightfold spatial cross-validation approach for Gigante site.
2. **cross-site spatial cross-validation,** where the models are trained on one site (eg. Gigante Peninsula) and validated on the other site (eg. Nouragues) and vice versa resulting in a twofold cross-validation approach.
3. **mixed-site spatial cross-validation,** where the models are trained and validated on data from both sites. For Gigante site, we split the two 15 by 15 m plots into six equal disjoint sub-areas of size 7.5 by 5 m each and for Nouragues, we did not split the plots and had six areas in total. As a results, we performed a sixfold cross-validation by using one sub-area from each of the plots in Gigante and one plot from Nouragues as validation set and the rest of the areas as training set as shown in Fig. 6

**Fig. 6:**
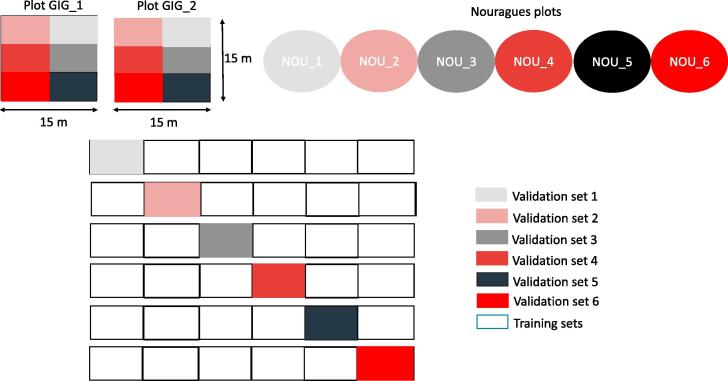
Mixed-site sixfold spatial cross-validation approach.

We chose these three different validation schemes to evaluate the performance of the method on different real-world usage scenarios. Self-site cross-validation evaluates the performance of the method in the base case scenario when new data comes from the same site as the data used for model building. Cross-site cross-validation indicates how the model would perform when the data comes from previously unseen site. Mixed-site cross-validation indicates the performance of the more sophisticated model, which has used data from multiple sites for building the model.

As liana woody point classification problem is an imbalanced classification problem with liana woody points belonging to the minority class, the traditional assessment metrics like accuracy and Receiver Operating Characteristics (ROC) curves may provide an overly optimistic view of the classifier performance (He and Garcia, 2008, Ozenne et al., 2015, Saito and Rehmsmeier, 2015). For instance, a classifier which always predicts a given point as a Class 2 point (leaves,tree or other vegetation) might yield an accuracy of 95% in a plot with only 5% liana woody points. As a result, to estimate the performance of the classifier in an unbiased manner, we used precision, recall, F_1_ score, precision-recall (PR) curves and False Positive Rate (FPR) as metrics using liana woody points as the positive class.

**Precision** quantifies the success of the classifier in predicting more lianas as lianas than non-lianas. It is the fraction of correctly classified liana points (True Positives (TP)) out of all classified liana points (TP and False Positives (FP))

(1) $$\mathrm{Precision}=\frac{\mathrm{TP}}{\mathrm{TP}+\mathrm{FP}}$$

**Recall** quantifies the success of the classifier in correctly predicting most of the liana points. It is the fraction of correctly classified lianas (TP) out of all lianas in the data (TP and False Negative (FN))

(2) $$\mathrm{Recall}=\frac{\mathrm{TP}}{\mathrm{TP}+\mathrm{FN}}$$

**F**_**1**_**score** is the harmonic mean of both precision and recall with 1 as its best value and 0 as its worst

(3) $$F_{1}\;\mathrm{score}=2*\frac{\mathrm{Precision}*\mathrm{Recall}}{\mathrm{Precision}+\mathrm{Recall}}$$

**Precision-Recall (PR) curve** shows the trade off between precision and recall for different liana woody point classification threshold values. The threshold values are derived from the probability with which the positive class (liana woody points in this case) is predicted by the model and could range from 0 to 100%. A very high threshold value indicates a model with very high precision but low recall and vice versa. As a result, a high area under the PR curve indicates a model with both high precision and recall. We use Average Precision (AP) to summarize the result of the PR curve. AP is calculated as the weighted average of precision at each threshold with the increase in recall from the previous threshold used as the weight. An ideal PR curve has a precision of 1 for every increase in recall resulting in an AP of 1.

**False Positive Rate (FPR)** quantifies the percentage of leaves, trees and other vegetation predicted as lianas. It is the fraction of Class 2 points (trees, leaves and other vegetation) misclassified as lianas (False Positives (FP)) out of all the Class 2 points in the data (FP and True Negatives (TN))

(4) $$\mathrm{FPR}=\frac{\mathrm{FP}}{\mathrm{FP}+\mathrm{TN}}$$

### 3.2 Classification workflow and post-processing

We explained above the steps involved in the development and assessment of RF model for extracting liana woody points from forest plots in the previous section.

In this section, we propose the following simple post-processing steps, which are an essential part of the processing pipeline (Fig. 1), to correct the misclassified liana woody points resulting from the RF model predictions. The misclassified points belong to two different categories: False Positives (FP) and False Negatives (FN). We propose four post-processing steps to correct these misclassified points. Steps 1 and 2 are mainly focused on removing FPs, Class 2 points that are misclassified as liana woody points, with basic filtering and with very minimal manual intervention as explained below. Steps 3 and 4 focus on correcting the FNs. This means that these steps mainly correct the liana woody points that are misclassified by the model as belonging to Class 2 (trees, leaves or other vegetation).

**Step 1** This step aims at filtering noisy or ghost points from the Class 1 (liana woody points) model predictions. We apply the Statistical Outlier Removal (SOR) filter implemented in the CloudCompare software (Girardeau-Montaut, 2011) to remove the noisy points that are too far from their neighbors in the point cloud classified as belonging to liana woody points by the model (Rusu and Cousins, 2011). SOR filter has two parameters: number of points to be considered for distance mean distance (nPoints) estimation and standard deviation multiplier threshold to be used for filtering the points (nSigma). We used the following default settings of the CloudCompare: nPoints  = 6 and nSigma  = 1.00

**Step 2** This step aims at removing the FPs from Class 1 model predictions. After SOR filtering, we manually remove the obvious sections belonging to Class 2 (points that are not liana woody components) from the resulting point cloud from Step 1 using the polygonal selection tool in CloudCompare (Girardeau-Montaut, 2011).

**Step 3** We correct the misclassified liana points in the Class 2 model predictions using the DBSCAN algorithm as follows. DBSCAN is a density-based clustering algorithm, which given a set of points in some space (Cartesian space in this case) groups together the points that are closely packed together. DBSCAN has two main parameters influencing the resulting clusters: ∊ and minPts. ∊ defines the maximum distance between two points to be considered to belong in the same cluster and minPts is the number of points to be in the neighborhood, defined by ∊, for a point to be considered as a core point. Once the core points are identified, the points that are connected to the core points in the ∊ neighborhood are identified and merged. For more information on DBSCAN, we refer to the original paper of (Ester et al., 1996).

We identify the isolated cluster of points among the points classified as Class 2 by the RF model. We achieve this by applying the DBSCAN on the predicted Class 2 points.

**Step 4** All the individual clusters of the predicted Class 2 points from Step 3 are then tested for connectivity with the corrected liana woody points from Step 2. First, we cluster the corrected liana woody points into small components using DBSCAN. We then test if a Class 2 cluster is in the ∊ neighborhood of any of the corrected liana woody point clusters (i.e. if these two clusters are close enough to be merged into a single cluster using DBSCAN).

We chose a value of 0.05 m for ∊ and 5 for minPts for both Step 3 and Step 4. The choice of ∊ here is mainly driven by the average point spacing in the point cloud. Since we downsampled our point cloud to have a point spacing of 0.04 m, we chose 0.05 m as our ∊ to ensure that only the densely connected points are retained.

## 4 Results and discussion

In section, we first report the results for hyperparameter optimization to build the RF model. Then, we report the results of the model performance for three different cross-validation categories explained above. Finally, we report the results of the post-processing steps at individual plot-level for the self-site cross-validation models.

### 4.1 Model development

The model performance was assessed by a fivefold cross-validation approach for hyperparameter optimization. The entire data from all eight plots in Table 1 was put together and then split into five random spatially disjoint sets, out of which four sets were used for training and one set was used for validation. The resulting F_1_ is the average result of all five model runs.

**Fig. 7:**
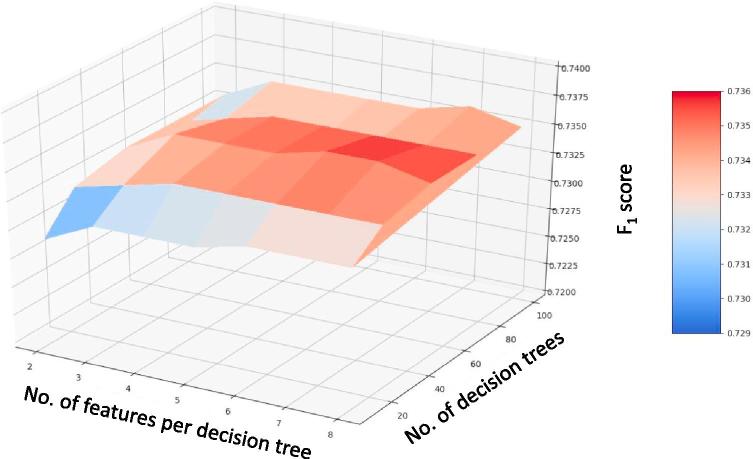
Illustration of the hyperparameter optimization having very little effect on the F_1_ score. The hyperparameters optimized are number of decision trees and the number of features to be chosen per decision tree.

The model performance remained relatively stable with F_1_ scores ranging between 0.72 and 0.73 for all different combinations of the two hyperparameters (Fig. 7). However, the F_1_ remained at 0.73 beyond 50 decision trees. As a result, we chose the number of decision trees as 50. The number of features did not seem to play a major role in the model performance and hence we chose the default value of $\sqrt{15}$ as suggested in (Breiman, 2001).

### 4.2 Predictive performance of random forest classifier

**Fig. 8:**
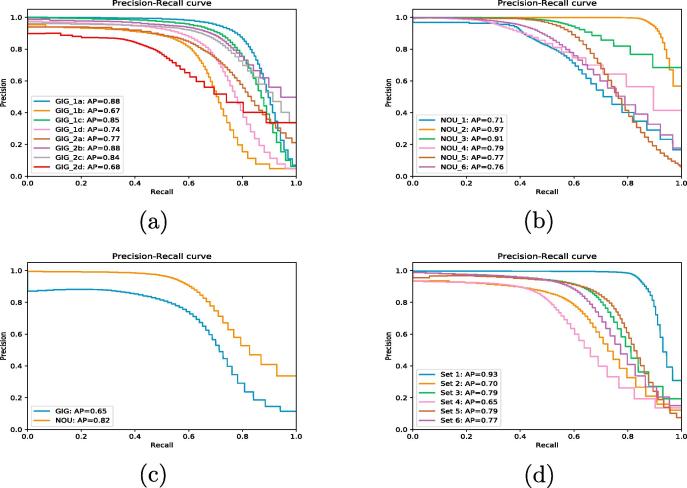
Precision-Recall curves for all three cross-validation categories. AP refers to the average precision explained in Section 3.1.5. (a) Self-site cross-validation: Gigante Peninsula. The plot names for each site are given in Fig. 5 for Gigante. (b) Self-site cross-validation: Nouragues. The plot names are given in Table 1 for Nouragues. (c) Cross-site cross-validation: GIG refers to the model built on Nouragues site and validated on Gigante site. NOU refers to the model built on Gigante site and validated on Nouragues site. (d) Mixed-site cross-validation: The name of the validation datasets in the figure are explained in Fig. 6.

We report all the performance metrics for all three cross-validation categories in Table 2 and show the PR curves for all them in Fig. 8. FPR was very low with values ranging between 1 to 3% across all three categories of spatial cross-validation.

**Table 2:** Precision, recall, F_1_ score, Average Precision (AP) and False Positive Rate (FPR) metrics for all three cross-validation categories. The results are the average of the eightfold and sixfold cross-validation for Gigante and Nouragues respectively for self-site category. The results are the average of the performance metrics from sixfold cross-validation shown in Fig. 6 for the mixed-site category. The standard deviation of the results are reported in the brackets. The overall average performance metrics of the random forest classifier is indicated as "Average" (in bold letters) in the last column of the table.

Cross-validation strategy	Dataset type	Precision	Recall	F_1_ score	AP	FPR
Self-site	Gigante Peninsula	0.85 (±0.06)	0.65 (±0.1)	0.73 (±0.1)	0.79 (±0.08)	0.03 (±0.02)
	Nouragues	0.91 (±0.09)	0.54 (±0.1)	0.66 (±0.2)	0.81 (±0.1)	0.01 (±0.004)
						
Cross-site	Train: Gigante Pen. and Val.: Nouragues	0.95	0.59	0.73	0.84	0.01
	Train: Nouragues and Val.: Gigante Pen.	0.82	0.51	0.63	0.65	0.01
						
Mixed-site	Mixed-site dataset	0.89 (±0.05)	0.62 (±0.12)	0.73 (±0.09)	0.77(±0.09)	0.01 (±0.004)
						
	**Average**	**0.88**	**0.58**	**0.69**	**0.78**	**0.014**

#### 4.2.1 Self-site cross-validation

The model performed with a high precision of 0.85 and 0.91 for Gigante and Nouragues respectively. However, the recall values were much lower for Nouragues compared to Gigante. This was mainly due to the thick straight liana stems (> 5 cm) having a geometrical structure similar to that of trees closer to the ground.

The high AP values from the PR curves of 0.79 and 0.81 for Gigante and Nouragues respectively indicate that the model has a good discriminatory power. The corresponding PR curves are shown in Fig. 8a and b. As it can be seen from the figure, precision retains a value of 80% while the recall is increased to 70% for most of the models resulting in the higher AP value.

#### 4.2.2 Cross-site cross-validation

Both the independent and average performance metrics of the cross-site cross-validation is shown in Table 2. Training on data from Gigante site and testing on Nouragues gives better results than both training on Nouragues and testing on Gigante and training and testing on Nouragues. This is in line with our expectations, as the plots GIG_1 and GIG_2 combined have more lianas then the plots in Nouragues resulting in more robust training data for the model to learn from.

Similarly the AP of the model trained with the data from Nouragues site is only 0.65, indicating a much lower discriminatory power than the model trained with data from Gigante site with a high AP of 0.84. The corresponding PR curves can be seen in Fig. 8c. As the PR curve indicates, the recall of the model drops to a low value of close to 60% for a recall of 70% explaining the lower AP value.

#### 4.2.3 Mixed-site cross-validation

We indicate the average performance metrics of the sixfold cross-validation (Fig. 6) in Table 2. As the results indicate, the model has a high precision of 0.89, a rather low recall of 0.62 and an F_1_ score of 0.73.

The PR curves indicate a good discriminatory power of the model in general with an AP of 0.77 (Fig. 8d).

### 4.3 Performance of the post-processing steps

**Fig. 9:**
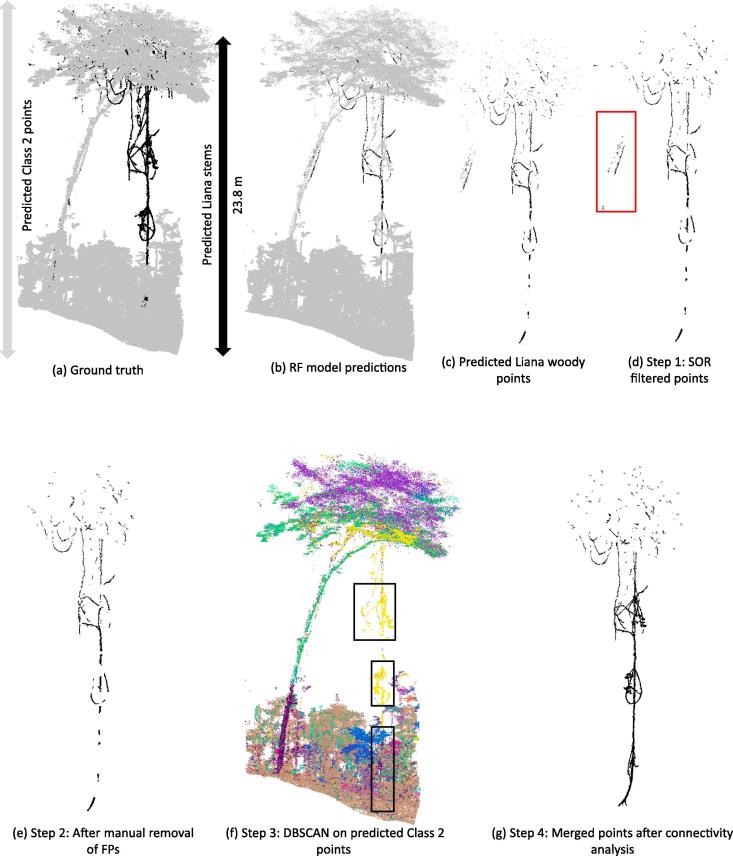
Illustration of the results from the post processing steps for the plot NOU_4. (a) Ground truth of the plot NOU_4 (b) Model predictions of the plot NOU_4. (c) Points predicted as liana woody points by the model. (d) Step 1 of post-processing: noisy points removed by Statistical Outlier Removal (SOR) filtering. (e) Step 2 of post-processing: points after manually removing the misclassified tree segments using CloudCompare (indicated by red squares in (d)). (f) Step 3 of post-processing: DBSCAN clustering algorithm on predicted Class 2 points. Different colors indicate points belonging to different clusters. (g) Step 4 of post-processing: Final liana woody points after merging the isolated clusters indicated by black squares in (f) with corrected points from (e). (For interpretation of the references to colour in this figure legend, the reader is referred to the web version of this article.)

The liana woody points can be extracted from the 3D data of a tropical rainforest plot by the final model trained on the entire dataset including all eight plots from two sites. As the results indicate, the models validated using the different cross-validation categories have on average high precision and very low FPR across all the plots from the two sites used in the study. This means that when the model predicts a point as a liana woody point, the model is 88% sure that its correct (indicated by the average from Table 2). This explains the very low FPR of 1.4% of the model. The model’s high predictive performance is further confirmed by analyzing the PR curves of the different models built. AP, which summarizes the PR curve of the models, is high with an average value of 0.78 indicating a good discriminatory power of the model. However the recall is rather low with an average value of 0.58. This means that only 58% of all liana woody points in the plot are retrieved by the model. The recall values are much lower especially for plots in Nouragues because of the thick straight stems having geometrical structures similar to trees closer to the ground. In addition, parts of liana tangles are mis-classified as they look like a cluster of points with geometrically similar features as leaves (Fig. 9a).

Since the FPR is much lower and is mainly caused by small stems that do not grow beyond a few meters from the ground and small segments of tree branches, we recommend a basic SOR filtering and manual removal of these small segments below the canopy as explained in Section 3.2. This manual removal is straightforward as it involves removing some isolated segments that belongs to trees. After the removal of FPs, we perform DBSCAN clustering and connectivity analysis to correct the FNs.

We report the increased recall from the post-processing steps on the predicted liana woody points from all the plots of Gigante Peninsula and Nouragues sites from the self-site cross-validation models in Table 3. While the cross-site models report the results for all the plots combined for a site and mixed-site models report the results for plots from the two sites combined, self-site models let us evaluate the effectiveness of the post-processing at individual plot-level.

**Table 3:** Increased recall for all eight plots from both Gigante Peninsula and Nouragues sites after applying the post-processing steps on the self-site cross-validation model predictions. Model recall for GIG_1 and GIG_2 are the average recall from the four small sub-plots of GIG_1 and GIG_2 respectively as shown in Fig. 5.

Site name	Plot Name	Model recall	Final recall after post-processing
Gigante Peninsula	GIG_1	0.69	0.73
	GIG_2	0.61	0.68
	**Average**	0.65	0.70
			
Nouragues	NOU_1	0.50	0.89
	NOU_2	0.84	0.87
	NOU_3	0.47	0.95
	NOU_4	0.28	0.98
	NOU_5	0.67	0.87
	NOU_6	0.46	0.82
			
	**Average**	0.54	0.90

The different post-processing steps are illustrated in Fig. 9.

**Fig. 10:**
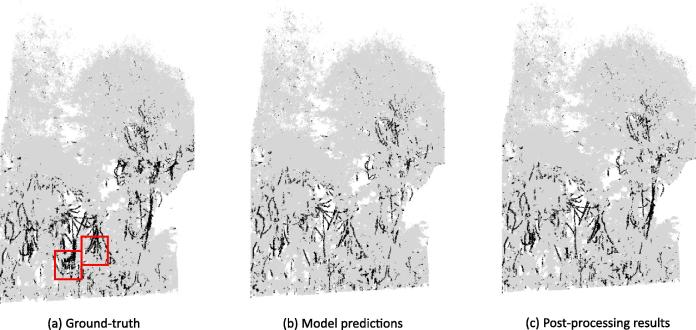
(a) Ground truth and (b) Model predictions for plot GIG_1 in Gigante (c) Post-processing result. Red squares indicate the areas which could not be corrected by post-processing. (For interpretation of the references to colour in this figure legend, the reader is referred to the web version of this article.)

These post-processing steps increased the recall from 54% to 90% in Nouragues plots without compromising on the precision. However, in Gigante the recall only increased by 5% from the original 65% resulting in a 70% recall overall. This was mainly because, the misclassified liana woody points were too close to the other vegetation to be considered as separate clusters as seen in Fig. 10. These 30% missing liana woody points could be manually corrected in a straightforward way using CloudCompare.

### 4.4 Liana extraction library

We provide an open source python software package executing the steps in Fig. 1 for the plot-level near-automated liana woody points extraction given only the xyz coordinates of the pointcloud. Extracting eigen values at multiple spatial scales on a small plot with approximately 500,000 points can be completed within 10 min even on a single core machine. However, this high computational efficiency comes at a cost of high memory requirements. The main specifications of the machine we used to run the algorithm are the following: dual boot (Ubuntu 16.04 LTS and Windows 10), Intel®  Xeon®  E5-1650 v4, 6 cores and 128 GB RAM. The python package has been tested on both Ubuntu and Windows operating systems. More details on the practical implementation and tips to work around the issue of high memory requirements of the algorithm can be found in the github page (https://github.com/sruthimoorthy/automated-liana-extraction.git).

### 4.5 Discussion

Our RF model performance indicates that the liana data is not site-specific as the model trained on Gigante site could be applied to data from Nouragues. The low F_1_ score of the model trained on Nouragues and tested on Gigante is mainly due to lack of sufficient training data as Nouragues had fewer lianas than Gigante. However, additional testing is needed on other sites to confirm this. The proposed post-processing steps increased the recall to 90% in Nouragues from a low value of 54% for the self-site cross-validation models mainly because of the very high precision of 91% of the models.

It is possible that an important source of error in the RF model development might arise from the errors made while manually labelling liana points vs non-liana points for training and testing data especially in the canopy. In the case of TLS, minimum size of the object distinguishable by the scanner increases with increasing distance from the scanner. For most of the lianas, their woody component becomes almost negligible in the canopy as they are much smaller than the trees even when they are closer to the ground (Dewalt et al., 2000). This is a sensor limitation and not a limitation of the method as such. Future development of TLS instruments with smaller beam divergence could help in resolving smaller objects at greater heights in the canopy.

The presented method is transferable to point clouds from any platform of similar high quality (i.e. minimal occlusion in the data). In addition, the proposed method only requires xyz coordinates of the points (refer to Fig. 1) and does not depend on any spectral properties of the points making it broadly applicable to 3D data collected from a variety of sensors. Another major advantage of the proposed method is combining the geometric features at multiple spatial scales, thus eliminating the need for optimal scale selection.

Future work could focus on improving the performance of the classifier (especially the recall value), thereby reducing the amount of manual intervention required, using more sophisticated machine learning methods like deep learning. Recent advances in the field of machine learning have extended the deep learning for 3D point cloud classification to work directly on the unstructured point clouds (as opposed to structured voxels etc.), thereby enabling automated feature learning to build the classifier (Qi et al., 2017). Though deep learning requires large amount of data and substantial computing power (high-performance GPUs), it is one of the promising ways to improve the classifier performance and will be a topic of our future study.

## 5 Conclusions

We presented a method for semi-automated liana extraction from plot-level TLS data with a high precision of 88%. The proposed method was tested on two sites: Gigante Peninsula, Panama and Nouragues, French Guiana. RF models had a low recall of 54% and 65% in Nouragues and Gigante, which was increased to 90% and 70% by the post-processing steps respectively. The method is semi-automated and the remaining 10 to 30% of the points requires manual intervention. Future developments of the method should focus on reducing this manual intervention by using more sophisticated methods like deep learning for liana woody point classification. We believe that our method will accelerate the research in high liana dense forests (e.g., Gigante Peninsula (van der Heijden et al., 2015)) and in forests where lianas continue to proliferate (Schnitzer and Carson, 2010, Schnitzer and Bongers, 2011, Schnitzer et al., 2000, Schnitzer, 2018), thereby enabling the quantification of their impact on tree and forest structure. The method enables researchers working on tropical forest plots to have a liana(noise)-free point cloud enabling an easier isolation of trees either manually or through an automated algorithm leading to an accurate quantification of tree structural metrics like tree volume. The presented method enables us to monitor and quantify liana abundance and biomass in forest plots, which is highly uncertain when calculated from allometric equations (Miao et al., 2016).

## Author contributions

S.M.K.M., Y.B. and H.V. conceived and designed the experiments with inputs from S.A.S. and K.C.; S.M.K.M. collected the data; S.M.K.M. and Y.B. performed the analysis; S.M.K.M. wrote the paper with critical contributions from all the authors.

## Data accessibility

All the codes required for extracting lianas in a plot are available in the following github page: https://github.com/sruthimoorthy/automated-liana-extraction.git. The codes are written in Python scripting language and have been tested in both windows and ubuntu. The data used for building the classifier can be obtained by sending an email to the corresponding author (sruthi.krishnamoorthyparvathi@ugent.be).

## Figures and Tables

**Fig. A.11:**
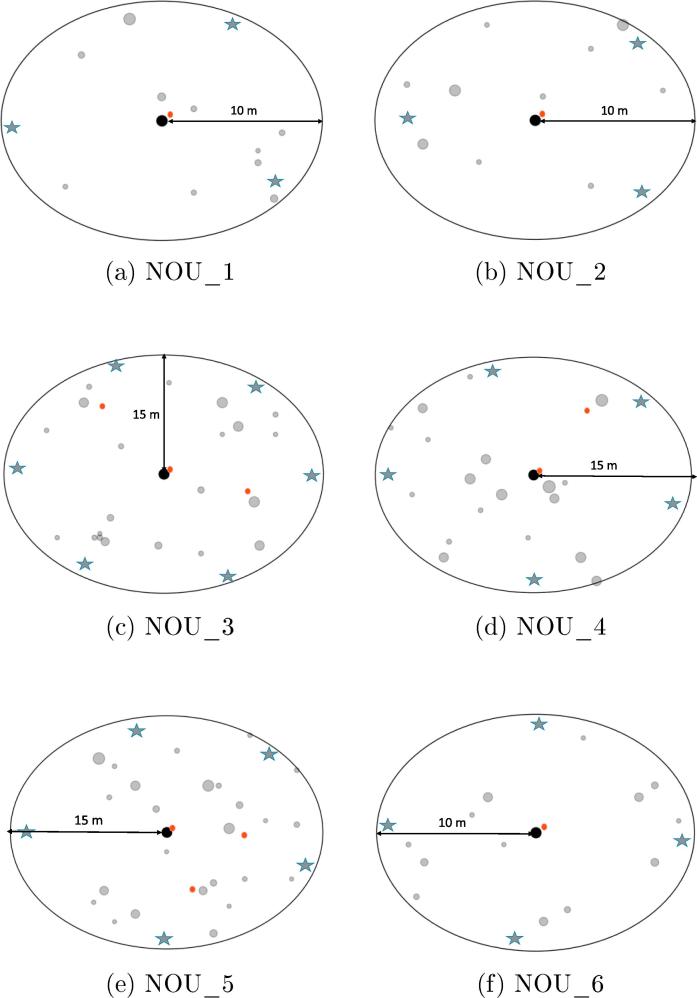
Scanning protocol for all the plots in Nouragues, French Guiana. ( Scan position,  target tree,  lianas in the plot and  other trees (> 10 cm) in the plot). Size of  and  circles reflects the relative size (DBH) of the tr.ees in the plot.

**Fig. A.12:**
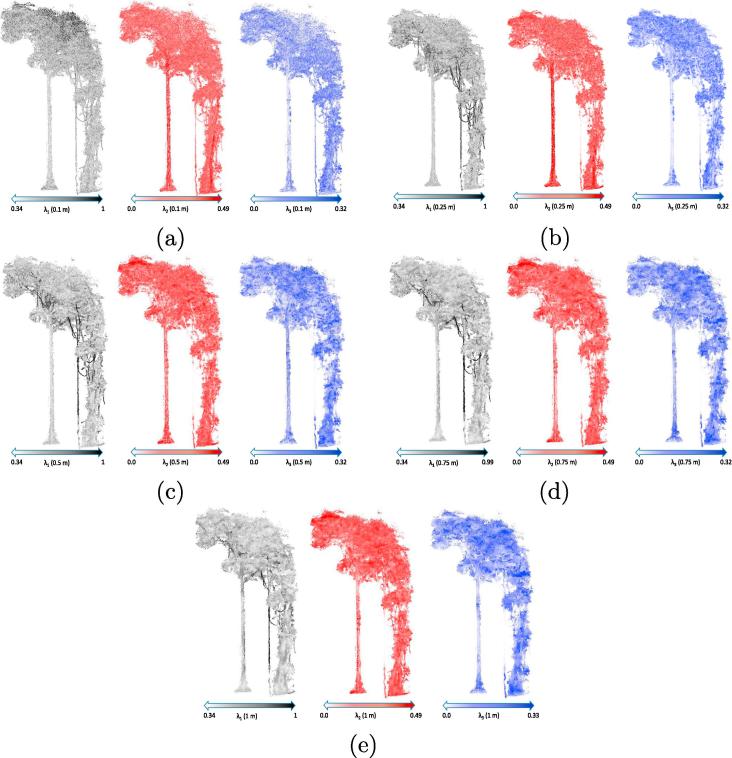
Illustrating the effectiveness of eigen values at multiple scales for classifying lianas in Nouragues (sample from plot NOU_5 in Table 1). The ground truth of the same plot is given in Fig. 3. The color scales at the bottom of each figure indicate the range of $\lambda_{1},\lambda_{2}$ and $\lambda_{3}$ at (a) 0.1 m, (b) 0.25 m, (c) 0.5 m, (d) 0.75 m and (e) 1 m spatial scale from left to rig.ht respectively.

## Appendix A

### A.1 Scanning protocol for plots in Nouragues

The scanning pattern for the six target trees in Nouragues is shown in Fig. A.11.

### A.2 Eigen value at muliple spatial scales for NOU_5 plot

The eigen values $\lambda_{1},\lambda_{2}$ and $\lambda_{3}$ for the NOU_5 at the spatial scales of 0.1, 0.25, 0.5, 0.75 and 1 m are shown in the Fig. A.12.
